# A Step in the Right Direction

**Published:** 1902-08

**Authors:** 


### A STEP IN THE RIGHT DIRECTION.

An exchange reports that there has recently been organized at Dayton, Ohio, a medical club, the membership of which is to be drawn from all schools recognized by the State. The object of this club is to discuss questions that are of importance to the general public welfare and to the profession. Dr. C. A. L. Reed, of Cincinnati, was the guest of honor at the first meeting and delivered an interesting address.

This is a step in the right direction, and it is to be hoped that similar clubs will be formed in other States. By thus acting together the profession will be able to secure the enactment of desirable medical laws and to bring about the adoption of many needed reforms in State and municipal hygiene. Because good men differ as to their therapeutic beliefs, there is no reason why they may not meet socially and work together in the interest of their own and the public welfare. R.

